# Orthodontically induced root resorption in endodontically treated and vital teeth: a cone beam computer tomographic study

**DOI:** 10.1186/s40510-025-00553-7

**Published:** 2025-02-27

**Authors:** Ziang Liu, Yuqing Ouyang, Yiting Lou, Yineng Han, Mengting Lu, Mengfei Yu, Huiming Wang, Wanghui Ding

**Affiliations:** 1 Stomatology Hospital, School of Stomatology, Zhejiang University School of Medicine, Hangzhou, China; 2 Zhejiang Provincial Clinical Research Center for Oral Diseases, Hangzhou, China; 3 Key Laboratory of Oral Biomedical Research of Zhejiang Province, Hangzhou, China

**Keywords:** External root resorption, Orthodontic treatment, Root canal treatment, Cone beam computed tomography

## Abstract

**Background:**

Orthodontically induced root resorption (OIRR) is a common side effect of orthodontic treatment. This study compares the degree of OIRR between root-filled teeth (RFT) and vital pulp teeth (VPT), and analyzes relevant study variables.

**Methods:**

We conducted a retrospective study on 69 patients who had undergone orthodontic treatment. Using Cone-beam computed tomography (CBCT), we measured changes of root length before and after treatment through a unique method involving three-dimensional (3D) image registration and superimposition. Factors related to the OIRR such as gender, type of treatment, tooth type, age, duration of treatment and distance of root movement were considered.

**Results:**

The sample included 55 females and 14 males aged 27.19 ± 6.08 years. On the basis that there was no significant difference in the root movement distance between RFT and VPT, RFT showed significantly less OIRR than VPT (*P* < 0.05). Gender did not significantly impact on OIRR for either RFT or VPT group (*P* > 0.05). In women specifically, RFT displayed less resorption than VPT (*P* < 0.05). For treatment type, extraction cases demonstrated a lower degree of OIRR in RFT than VPT (*P* < 0.05), and notable greater OIRR in with-extraction group compared to no-extractions group was found in RFT (*P* < 0.05), but not in VPT (*P* > 0.05). Tooth type did not yield significant differences in OIRR overall; however, upper teeth and premolars experienced lower resorption in RFT than in VPT (*P* < 0.05). Cases treated with fixed appliance had higher OIRR in both RFT and VPT than those with clear aligners (*P* < 0.05). Age did not correlate significantly with OIRR for either group (*P* > 0.05). And duration of treatment positively correlated with OIRR for both types (RFT: r = 0.5506, *P* < 0.0001; VPT: r = 0.4371, *P* = 0.0002), so did root movement distance (RFT: r = 0.2955, *P* = 0.0140; VPT: r = 0.2790, *P* = 0.0206).

**Conclusions:**

RFT exhibit significantly less OIRR than VPT after orthodontic treatment. Treatment type, appliance type, duration of treatment and root movement distance are significant factors influencing OIRR. Personalized orthodontic treatment plans and vigilant monitoring are crucial to mitigate OIRR risks.

**Supplementary Information:**

The online version contains supplementary material available at 10.1186/s40510-025-00553-7.

## Introduction

Orthodontically induced root resorption (OIRR) is a common side effect of orthodontic treatment, often resulting in tooth damage and potential loss. Also known as external apical root resorption (EARR), it’s challenging to predict or prevent [1, 2]. During orthodontic treatment, OIRR affects up to 90% of the teeth histologically. And radiographically, mild to moderate resorption appears in 48–66% of cases, while severe resorption requiring intervention occurs in 1–5% of cases. [1]

OIRR is linked with aseptic inflammation of periodontal tissues under pressure leading to changes like hyaline degeneration [3]. Excessive stress can cause the apoptosis of cementoblasts, exposing the root surface to odontoclastic attack mainly at the apical region [4]. This process involves interactions among osteoblasts, osteoclasts, odontoblasts, and odontoclasts regulated by cytokines and hormones. [5, 6]

The causes behind OIRR are complex involving biological factors such as genetics, nutrition etc., along with mechanical ones like force magnitude during orthodontic treatment [7–10]. Individual variations are significant especially for patients with root-filled teeth (RFT). Recent studies [6, 11, 12] suggest lower OIRR rates in RFT compared to vital pulp teeth (VPT), but these relied on panoramic radiographs, indicating a need for further evidence-based investigation.

Traditional assessments using periapical and panoramic radiographs [13–19] face certain challenges such as geometric distortion, overlapping teeth, and poor visualization of root structures, resulting in incorrect landmark identification and reducing measurement accuracy [20]. Cone-beam computed tomography (CBCT) overcomes these limitations by providing high-resolution, distortion-free images effective for detecting minimal degrees of OIRR [21, 22]. Lima et al [23] demonstrated CBCT's superiority in diagnosing root resorption in endodontically treated teeth compared to periapical radiography. Castro et al [24] were pioneers in adopting CBCT to measure teeth with filled roots or vital pulp at their maximum long axial length from root apex to corresponding cusp tip. This innovative approach, however, may not fully account for the impact of crown wear, thus not reflecting the absolute change in actual root length. Grissom et al [25] utilized CBCT technology for three-dimensional imaging, offering a quantitative data analysis of teeth by calculating changes in tooth surface area and volume. This method contributes to a more objective evaluation of the impact of orthodontic treatment on teeth. Our study aims to build on these foundational works to evaluate the degree of OIRR between endodontically treated teeth versus their contralateral vital ones with increased sample size, while revealing OIRR influencing factors through multifactorial analysis.

## Material and methods

This retrospective clinical study utilized a split-mouth design to assess patients who had undergone orthodontic treatment at the Department of Orthodontics, Zhejiang Stomatological Hospital from May 2017 to September 2024. Since this is a retrospective study, Informed Consent is not applicable. Institutional Review Board (IRB) approval was obtained (Approval No. 2024–005).

### Inclusion criteria

1. Healthy patients without systemic diseases.
2. Adult patients (ages 18+).
3. Orthodontic treatment duration exceeding one year. And patients were treated with either 0.022’’ × 0.026’’ passive self-ligating bracket appliance or clear aligner (Invisalign, Align Technology, Santa Clara, Calif).
4. Complete orthodontic records and high-quality CBCT data.
5. No previous orthodontic or orthognathic treatments.
6. At least one root canal-treated tooth before orthodontics with radiographs showing dense root canal filling material located 0.5 to 2.0 mm from the apex, alongside its contralateral vital tooth.
7. Endodontically treated tooth monitored for at least 1 year, and both root-filled and vital teeth had radiologically normal periapical tissues and no clinical signs or symptoms (like pain, swelling, fistula, crown discoloration, percussion, looseness, etc.).
8. Absence of untreated caries.
9. Periodontal probing depth less than 3 mm.

### Exclusion criteria

1. Craniofacial anomalies.
2. History of trauma, root fracture, bruxism, or congenital malformation in the teeth (like small teeth, abnormal position, excessive torsion, root deformity, etc.) in RFT and VPT.
3. Root resorption greater than 2 mm before orthodontics.
4. Teeth that were replanted or transplanted.
5. Incomplete root canal treatment of RFT (underfilling, overfilling or over-extension).
6. Orthodontics before orthognathic surgery.
7. Periodontal probing depth more than 3 mm.
8. Severe immune systemic disease such as allergy and asthma, and metabolic disease like diabetes.

### Image analysis

CBCT images were acquired before orthodontic treatment (T1) and at orthodontic debonding (T2) using a NewTom VGi scanner (Aperio, Milan, Italy) set as follows: 15 × 15 × 15 cm^3^ field of view, 110 kVp, 2 mA, and voxel size of 0.3 mm. Patients were positioned with the Frankfort horizontal plane (FHP) parallel to the floor and the teeth were placed in centric occlusion. The CBCT images were saved in DICOM format for analysis on 3D Slicer software 5.2 (open-source software http://www.slicer.org).

Before measuring root length, images at T1 and T2 were imported into ITK-SNAP 4.0 (open-source software http://www.itksnap.org) for registration and superimposition on the anterior cranial fossa using an automated voxel-based registration method according to Romano [26]. The images were then imported into 3D Slicer.

The measurement method utilized the principle of “overlapping images”. Based on volumetric data registration of the same tooth in T1 and T2 CBCTs, the distance of root apical points represented changes in root length. This overcame the limitation of unstable measurement planes when measuring root length on the dental root axis plane.

**Fig. 1:**
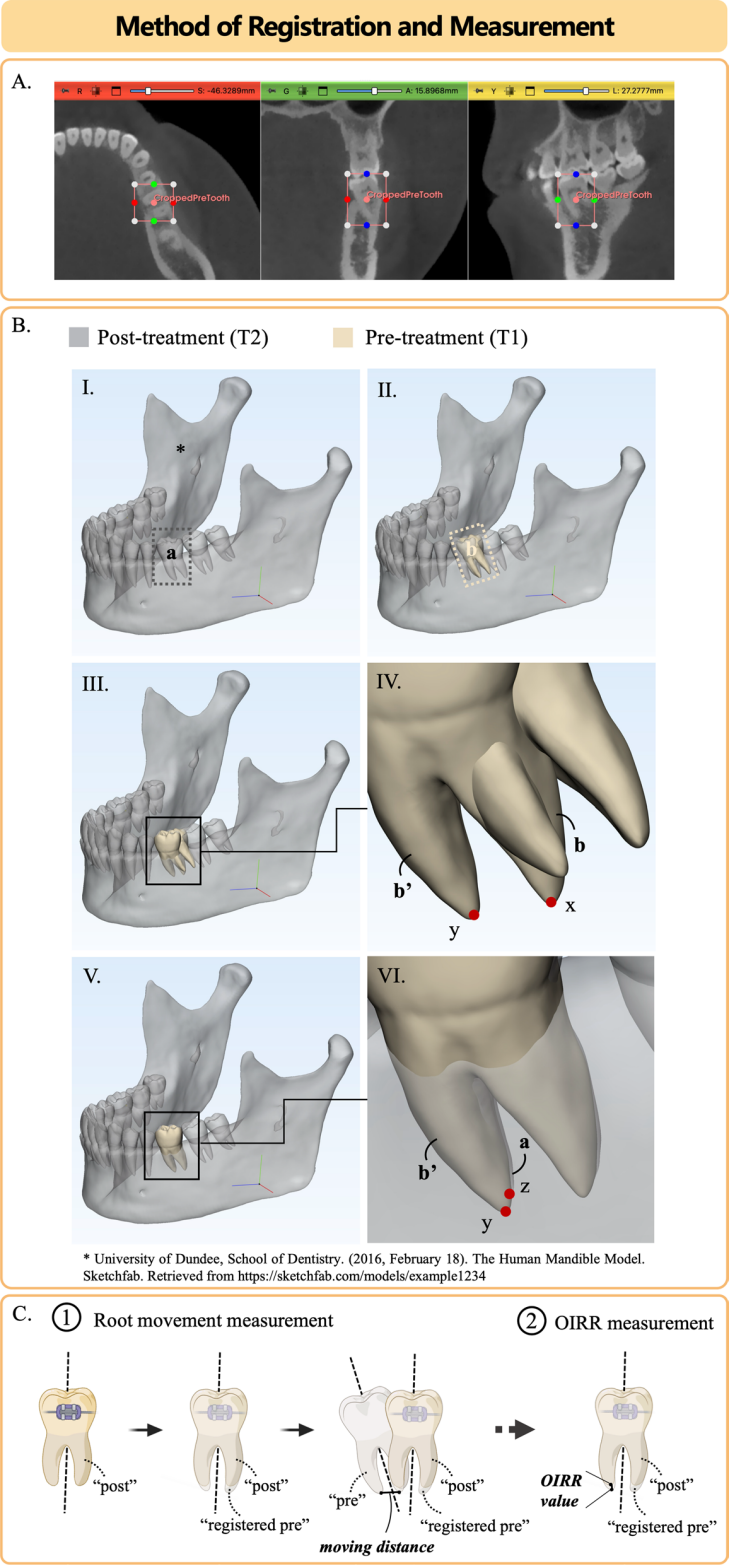
Method of Registration and Measurement. **A** A ROI including the entire roots of the target tooth with least irrelevant image information; **B**, **C** The schematic diagram of the measurement principle of OIRR and root movement distance. **I** Reconstruction of the mandible and the target tooth (taking mandibular left first molar as an example) according to the T2 CBCT; **II** Spatial position relationship of the target tooth after 3D superimposition on cranial base of T1 and T2 CBCTs. **a** Cropped volume of the target tooth from T2 CBCTs (CroppedPostTooth); **b** Cropped the volume of the target tooth from T1 CBCTs (CroppedPreTooth). **III, IV** Transform CroppedPreTooth (b) into RegCroppedPreTooth (b’) by rigidly registered to CroppedPostTooth (a) to to restore the original length of the tooth root at the post-treatment position. Take the mesial root as an example. x: apical point of CroppedPreTooth (b); y: apical point of RegCroppedPreTooth (b’). root movement distance = the distance between x and y. **V**, **VI** z: apical point of CroppedPostTooth (a). OIRR = the distance between y and z

Firstly, a region of interest (ROI) was created to include the entire root of the target tooth, minimizing irrelevant image information (Fig. 1A). A new volume containing only the target tooth was created using the “Crop Volume” module based on the ROI created earlier, resulting in volumes named CroppedPreTooth and CroppedPostTooth for each patient. Navigate to the "Registration" module and select the "General Registration (ANTs)" method. Register the volumes using CroppedPostTooth as the fixed image and CroppedPreTooth as the moving image. Choose "Rigid" from Stages (Presets). The output will be a Transformed Volume named RegCroppedPreTooth.

The distance between the root apex points of the tooth on CroppedPostTooth and RegCroppedPreTooth represented the amount of apical root resorption. And the distance between the root apex points of the tooth on CroppedPreTooth and RegCroppedPreTooth was measured and recorded as the root movement distance. For teeth with multiple roots, each root was separately assessed and the average was taken. The schematic diagram of the measurement principle is shown in Fig. 1B, C.

**Fig. 2:**
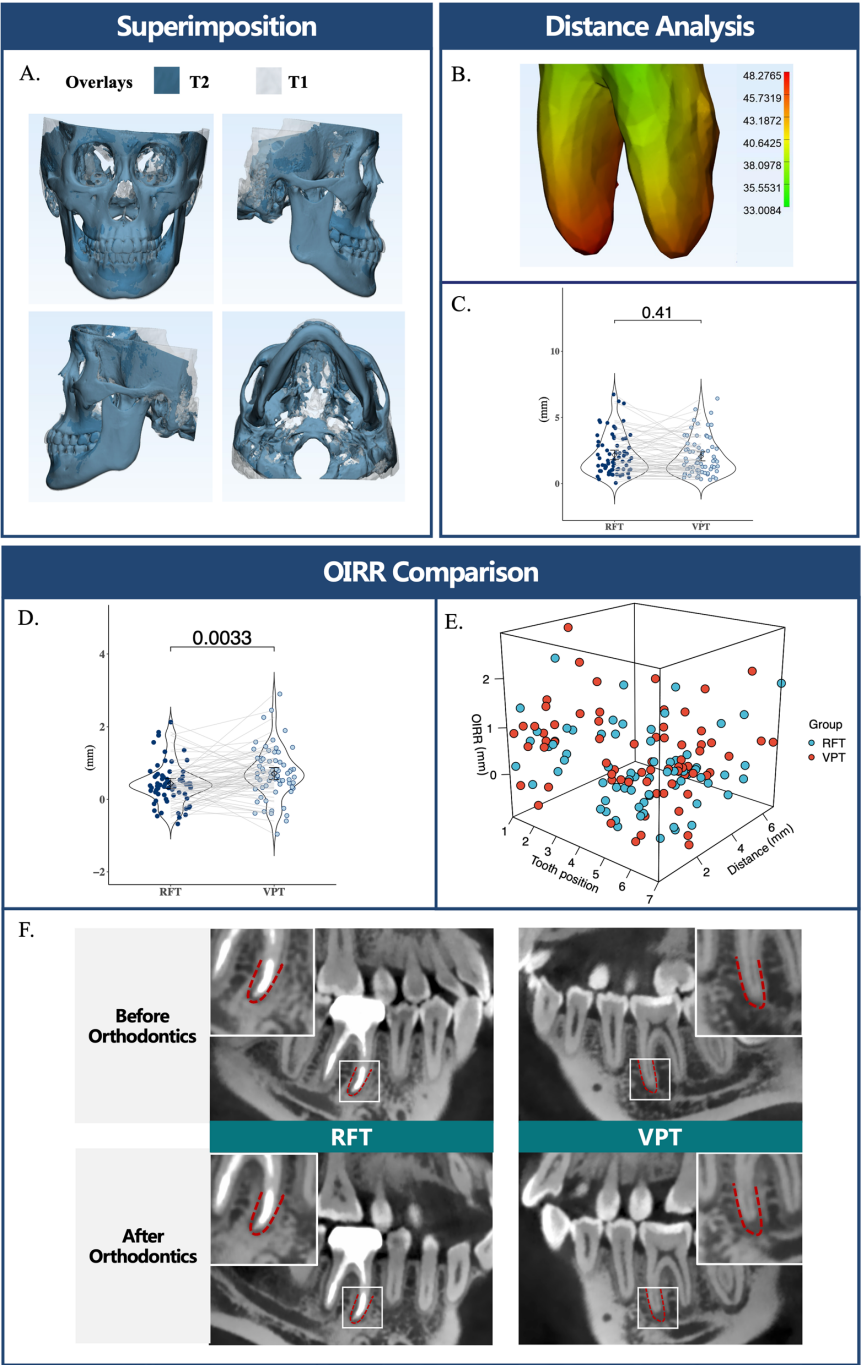
**A** Semitransparent overlay of T1 (shown in white) and T2 (shown in blue) CBCT images of one patient after being superimposed on the anterior cranial fossa; **B** Distances distribution of root surfaces between the two models 3d-reconstructed from T1 and T2 CBCTs shown by heat map; **C** The comparison of root apex moving distance between two groups; **D** Sample distribution by OIRR in RFT and VPT; **E** a 3D scatter plot visually represent the relationship between OIRR and tooth movement distance by tooth position order; **F** One case demonstrated that root resorption was lower in root-filled teeth than its contralateral vital pulp teeth after orthodontics

And Root movement distance was compared between RFT and VPT groups before OIRR of the two was compared. Figure 2A shows the semitransparent overlay of T1 and T2 CBCT images after being superimposed on the anterior cranial fossa showed well-superimposition, facilitating accurate measurement of root movement distance. Heat maps revealing the distances of root surfaces between the same tooth reconstructed from T1 and T2 CBCTs highlighted the apical region as the primary site of root change after orthodontics (Fig. 2B). Hence we adopted apical moving distance to represent the root movement distance, which didn’t show a significant difference between RFT and VPT groups (*P* > 0.05) (Fig. 2C).

The relationship between OIRR and contributing factors such as gender, age, tooth type (including incisor, premolars and molars), tooth position (upper teeth or lower teeth), treatment type (whether teeth were extracted), appliance type (fixed appliance or clear aligner), orthodontic treatment duration, and root movement distance were evaluated.

### Statistical analysis

Sample size calculation employed G*Power (version 3.1; Kiel, Germany). The effect size, as determined by difference of OIRR between RFT and VPT groups (− 0.28 ± 0.75 mm), was − 0.3733. A post hoc analysis was carried out with a significance level α set at 0.05 and sample sizes of 69 for both RFT and VPT groups, resulting in a statistical power of 0.86, indicating strong statistical power.

The ShapiroWilk test assessed normality, and Levene’s test assessed variance equality. Paired samples t-test compared root length changes between T2 and T1 among RFT and VPT groups. Paired t-test compared root apex movement distances between two groups. Linear regression examined the relationship between root change and tooth movement. The Student t-test or Wilcoxon test, Anova test or Kruskal test, and the Pearson correlation analysis for relevancy between dependent variables (OIRR in RFT, OIRR in VPT, difference in OIRR (dOIRR)) and contributory factors were applied. Statistical analysis was performed using the R 4.1.1 software (open-source software https://www.r-project.org/). For all data presented in this manuscript, *P* < 0.05 was recognized as a statistically significant difference based on a recent recommendation. The same clinician (Z.A.L.) performed all assessments of root resorption.

## Results

After applying the inclusion and exclusion criteria, 69 patients (55 females, 14 males; mean age 27.19 ± 6.08 years) were included. Both RFT and VPT groups consisted of 19 incisors, 15 premolars, 35 molars. The duration of orthodontic treatment exceeded 1 year for each patient (30.30 ± 10.65 months). And OIRR was found to be significantly lower in RFT compared to VPT (0.44 ± 0.57 mm vs. 0.67 ± 0.73 mm, *P* = 0.0033, Paired t-test) (Fig. 2D). We used a 3D scatter plot to visually represent the relationship between OIRR and tooth movement distance by tooth position order (Fig. 2E) and selected a typical case to demonstrate the difference in OIRR between the RFT and VPT (Fig. 2F). The correlation analyses between the dependent variables (OIRR in RFT, OIRR in VPT, difference in OIRR) and the independent explanatory variables (gender, treatment type, tooth type, tooth position and appliance type) are presented in Table 1.

**Table 1:** Correlations between the dependent variables (OIRR in RFT, OIRR in VPT, difference in OIRR) and contributory factors (gender, treatment type, tooth type, tooth position, and appliance)

		n	OIRR in RFT (mm)	OIRR in VPT (mm)	Difference in OIRR (mm)	*P* value
Total		69	0.44 ± 0.57	0.67 ± 0.73	− 0.28 ± 0.75	0.0033*
Gender	M	14	0.54 ± 0.71	0.76 ± 0.78	− 0.22 ± 0.69	0.2593
	F	55	0.41 ± 0.53	0.70 ± 0.67	− 0.29 ± 0.77	0.0072*
	*P* value		0.3434^‡^	0.7977	0.7358	
Treatment type	Non-extractions	33	0.29 ± 0.53	0.53 ± 0.70	− 0.24 ± 0.73	0.0685
	With extractions	36	0.57 ± 0.58	0.88 ± 0.65	− 0.31 ± 0.78	0.0229*
	*P* value		0.0112*^‡^	0.0848^‡^	0.7052	
Tooth type	Incisor	19	0.51 ± 0.56	0.81 ± 0.84	− 0.30 ± 0.90	0.1579
	Premolar	15	0.19 ± 0.60	0.70 ± 0.52	− 0.51 ± 0.86	0.0370*
	Molar	35	0.50 ± 0.55	0.66 ± 0.67	− 0.16 ± 0.60	0.1244
	*P* value		0.1076^§^	0.6165^§^	0.3170	
Tooth position	Upper	35	0.34 ± 0.50	0.68 ± 0.69	− 0.34 ± 0.70	0.0074*
	Lower	34	0.53 ± 0.63	0.74 ± 0.70	− 0.21 ± 0.80	0.1352
	*P* value		0.1422^‡^	0.7324	0.4823	
Appliance	Fixed appliance	56	0.53 ± 0.57	0.84 ± 0.64	− 0.31 ± 0.74	0.0026*
	Clear aligner	13	0.02 ± 0.36	0.14 ± 0.62	− 0.12 ± 0.80	0.6016
	*P* value		0.0011*^‡^	0.0019*^‡^	0.4403	

Data expressed as means ± standard deviations. M, male; F, female. *P < 0.05

Two-group analyses were performed using the t-test or ^‡^Wilcoxon test;

Multi-group comparisons were conducted using ANOVA or the ^§^Kruskal–Wallis test

Gender showed no statistically significant impact in OIRR in either RFT or VPT group (*P* = 0.3434 and *P* = 0.7977, respectively), nor in the OIRR difference between genders (*P* = 0.7358). However, females exhibited a slightly greater dOIRR, with OIRR of RFT statistically lower than VPT in women (*P* = 0.0072), while no such difference was observed in men (*P* = 0.2593). In terms of treatment type, while the no-extractions group showed lower OIRR in RFT compared to with-extractions group (*P* = 0.0112), this difference did not reach statistical significance in VPT (*P* = 0.0848). And only with-extractions subgroup exhibited lower OIRR in RFT than VPT (*P* = 0.0229). There exists no significant differences in different tooth types (incisors, premolars, and molars) in OIRR for RFT (*P* = 0.1076) and VPT (*P* = 0.6165), nor the OIRR differences (*P* = 0.3170). And premolars exhibited significantly lower OIRR in RFT than VPT (*P* = 0.0370). Tooth position comparisons revealed that there was no significant difference of OIRR between upper and lower teeth in both RFT and VPT groups (*P* = 0.1422 and *P* = 0.7324, respectively), whereas only upper teeth showed lower OIRR in RFT than VPT (*P* = 0.0074). Finally, appliance type might be a significant factor, with fixed appliance cases associated with a notably higher degree of OIRR in both RFT (*P* = 0.0011) and VPT (*P* = 0.0019) than clear aligner cases. Also, cases treated with fix appliances exhibited a significantly lower OIRR in RFT than in VPT (*P* = 0.0026), whereas clear aligners showed no significance (*P* = 0.6016).

The correlations between OIRR and variables such as age, treatment duration, and root movement distance were analyzed (Table 2). Age was not significantly correlated with OIRR in either RFT (r = −0.1377, *P* = 0.2592) or VPT (r = −0.0504, *P* = 0.6809). However, age showed a strong correlation with the dOIRR between RFT and VPT (r = 0.8362, *P* = 0.0254). Treatment duration showed a positive correlation with OIRR in both RFT (r = 0.5506, *P* < 0.0001) and VPT (r = 0.4371, *P* = 0.0002), suggesting that longer treatment duration might be associated with increased root resorption in both tooth types. However, no significant association was found between treatment duration and the dOIRR between RFT and VPT (*P* = 0.6009). Root movement distance also demonstrated a slightly significant positive correlation with OIRR in both RFT (r = 0.2955, *P* = 0.0140) and VPT (r = 0.2790, *P* = 0.0206), supporting the notion that greater root movement might lead to increased resorption. These findings highlight the impact of treatment duration and root movement distance on OIRR, while the role of age appears to be more complex, influencing only the dOIRR. A heatmap made by R package ggplot2 helped visualize data better and extract more information (Supplement Fig. 1).

**Table 2:** Correlations between the dependent variables (OIRR in RFT, OIRR in VPT, difference in OIRR) and contributory factors (age, treatment duration and root movement distance)

		OIRR in RFT (mm)	OIRR in VPT (mm)	Difference in OIRR (mm)
Age^**†**^	r	− 0.1377	− 0.0504	0.8362
	*P* value	0.2592	0.6809	0.0254*
Treatment duration^**†**^	r	0.5506	0.4371	− 0.0641
	*P* value	< 0.0001*	0.0002*	0.6009
Root movement distance^**†**^	r	0.2955	0.2790	
	*P* value	0.0140*	0.0206*	

^**†**^Spearman correlation. r, Pearson correlation coefficient; *P < 0.05

## Discussion

OIRR is an unpredictable and challenging issue to fix. The relationship between endodontics and orthodontics in the planning stage and the impact of orthodontic treatment on RFT remain uncertain. In our research, we introduce a novel measurement method that employs 3D image registration and superimposition of CBCT images for accurate measurements of changes in root length, providing technical support for further researches. Our study reveals that OIRR is more weaker in RFT than VPT, following previous meta-analyses [6, 11, 12, 27]. And it aligns with some literature [19, 28] suggesting a modified response to orthodontic forces in RFT due to the absence of vital pulp, which could potentially affect the resorption process.

VPT with active pulp inflammation and degeneration resulted from neurovascular disturbances are more prone to OIRR [29, 30]. This process is driven by molecular signaling systems that cause various tissue changes. Injured and stretched pulp cells release inflammatory cytokines like macrophage colony stimulating factor (M-CSF) and receptor activator of nuclear factor kappa B ligand (RANKL), which triggers odontoclastic activity [31]—an event that wouldn't happen without the presence of pulp. Neuropeptides released from the pulp play a role in root resorption, and their absence in RFT may reduce this risk [32]. Lower values of OIRR for RFT may be attributed to the modern endodontic treatment materials like ethylene diamine tetraacetic acid (EDTA) and calcium hydroxide. They create an alkaline environment [33, 34], inhibiting collagenase and acid hydrolase activity involved in root resorption and stimulating alkaline phosphatases for hard tissue repair [35, 36]. These materials also expose the extracellular matrix, facilitating cell adhesion and the release of bioactive molecules that aid cell differentiation and proliferation [37]. This promotes the migration of stem cells towards damaged root surface, initiating repair processes. Endodontically treated teeth show a slight edge in resisting OIRR or aiding its repair, suggesting that OIRR may not be a major concern during orthodontic treatment planning for RFT. However, this advantage is minor, so endodontic treatment should not be applied solely for managing external resorption in vital teeth but reserved for contaminated teeth or those with pulp necrosis to eliminate periapical inflammation [38]. When OIRR is detected, it is recommended to assess force distribution, movement intensity, and other factors within the scope of orthodontic treatment.

Several notable findings emerge regarding factors such as gender, treatment type, tooth type, tooth position, appliance, age, treatment duration, and root movement distance influencing OIRR in RFT and VPT.

Our findings indicate that gender does not directly influence the extent of OIRR within RFT or VPT independently, aligning with previous literature [39]. However, gender does appear to accentuate dOIRR between RFT and VPT in females. This effect may arise because females seem more sensitive to variations in pulpal status, possibly reflecting unique biomechanical or biological adaptations to orthodontic force. Further research is needed to explore these potential mechanisms. It is also possible that the relatively small sample size of males in our study limited our ability to detect significant differences within this subgroup.

Changes in root and alveolar bone with age have always been subjects of interest. Older patients tend to have narrower periodontal ligament spaces and denser alveolar bone, which may give rise to more frequent root resorption. In our study, however, there was no significant correlation between age and OIRR for either RFT or VPT (*P* > 0.05), consistent with findings from Alves’s meta-analysis [11]. Our results also indicate that as individuals get older, dOIRR may become more pronounced, implying a positive correlation between age and the impact of root canal treatment on OIRR.

Tooth type analysis showed no significant differences in OIRR among incisors, premolars and molars. However, there are differing views about specific tooth type susceptibility towards OIRR and incisors seem more prone perhaps due to their single-root morphology [40]. Furthermore, maxillary central incisors are most commonly affected by OIRR according various studies citing factors like close positional relationship among roots, the surrounding bone structures and common depression and adduction movement types [41, 42]. Furthermore, our findings suggest that the impact of root canal treatment on OIRR is more pronounced in premolars, though further research with a larger sample size is necessary to verify this effect across tooth types.

Our study also supports that fixed appliances tend to exert forces leading to greater root resorption compared to clear aligners, consistent with findings by Aldeeri, Fang and Li [43–45]. This could be an important consideration for minimizing OIRR in patients undergoing orthodontic treatment. However, our sample of cases involving clear aligners is relatively small, and additional data will be essential to further explore the impact of appliance type on OIRR.

The root movement distance partly reflects the duration of osteogenesis and osteoclasty activities in periodontal tissue, thus it’s somewhat related to root resorption. Wan et al.’s measurement of maxillary central incisors’ movement on the sagittal plane didn’t fully capture the actual moving distance because it was two-dimensional [46]. However, 3D reconstruction and visualization using CBCT images provide detailed information about root movements. Our study is pioneering in measuring real apical movement at a three-dimensional level by superimposing pre- and post-treatment CBCTs. In our study, no statistically significant difference was found between VPT and RFT regarding root movement distance, implying similar conditions for orthodontic tooth movement and making the results of OIRR extent comparison between the two groups more convincing. Our findings indicate that extensive root movement has an effect on OIRR degree in both RFT and VPT. And tooth extraction in orthodontic treatment can cause more severe root resorption due to longer moving distances, as supported by Supplement Fig. 2.

Longer treatment durations correlate positively with OIRR in both RFT and VPT in our research. This could be due to teeth moving over extended periods across greater distances and potential reciprocating tooth movement- highlighting efficient treatment planning importance and timely interventions for mitigating resorption risks.

In summary, our findings underscore complex factors influencing OIRR, including treatment type, appliance, treatment duration and root movement distance. Additionally, variables such as gender, treatment and tooth type as well as appliance contribute to variations in OIRR between RFT and VPT, making these factors particularly noteworthy when comparing the two groups. These insights can guide clinical decisions aimed at minimizing root resorption risk by considering patient-specific factors such as treatment type and appliance selection.

Additional pathogenic factors influencing OIRR include unreported trauma and apical periodontitis [47], systemic diseases and hormonal imbalances. Recent studies emphasize molecular and genetic factors such as interleukin-1β polymorphisms and osteopontin’s association with OIRR. However, while interleukin-17A was reported to stimulate odontoclastogenesis, no significant correlation was found by Linhartova et al. [48] And biomechanical factors contribute 10–30% to root resorption variation [49, 50]. Treatment duration, appliance type, and tooth extraction are recognized contributing factors to OIRR, but quantifying the impact of force magnitude and tooth movement pattern is more challenging. Yamamoto et al [51] indicated that light forces could decrease root resorption risk in rats, though the underlying mechanisms remain unclear. Currell et al [9] found heavy, continuous, and intrusive forces positively correlate with OIRR, with maxillary incisors being particularly susceptible [40, 52]. Similarly, Yamaguchi’s study indicated that continuous force leads to higher cytokine release and greater root resorption than intermittent forces [53]. In conclusion, mechanical and biological factors collectively influence OIRR [54], which underscores the need for further research to enhance treatment results.

## Limations

While this study provides some new findings, it does have several limitations. For example, we intended to measure OIRR at a volumetric level with thresholding techniques for three-dimensional image [55], but scatter and beam hardening artifacts in large-volume CBCT scans hindered this aspect of the research. Besides, the irregular curve of the tooth root surface after absorption means that using only the "root apex point" as a measurement index may not provide the most precise assessment. Measuring the curvature of the root apex would offer a more accurate assessment of tooth root absorption levels. Besides, although our study included a larger sample size compared to previous studies on similar topics, further expansion of the sample size in specific subgroups is still needed, particularly cases involving canines and cases treated with clear aligners. And its retrospective design limited the exploration of certain influencing factors like orthodontic loading regimes, while utilizing technologies like finite element analysis to simulate impact of distinct orthodontic forces on different tooth morphologies could enhance treatment planning and predictive capabilities. Also, longitudinal studies are recommended to observe the progression of OIRR over extended post-treatment periods for better understanding long-term impacts. In future studies, prospective studies with more case samples precisely designed and controlled for confounding factors are encouraged, to further examine different tooth types separately, and investigate the correlations between appliance type, jaw type (maxillary or mandibular), and OIRR. And relevant animal studies are needed to confirm the results and further explore the underlying mechanisms of this phenomenon.

## Conclusions

Within the limitations of this study, it was concluded that:

1. RFT exhibit significantly less OIRR than VPT after orthodontic treatment, indicating that the absence of vital pulp in RFT may lead to a unique biological response to orthodontic forces that mitigates the risk of OIRR.
2. Key factors influencing OIRR include treatment type, appliance type, treatment duration, and root movement distance, which can guide clinical decisions aimed at minimizing root resorption risk by consideration of treatment type and appliance selection. Notably, treatment duration positively correlates with OIRR in both RFT and VPT, underscoring the need for efficient treatment planning and timely intervention to mitigate resorption risks.

While RFT demonstrate a lower OIRR risk than VPT, careful consideration of individual patient factors and treatment parameters remains essential in orthodontic planning. Clinicians should be vigilant for signs of OIRR, particularly in VPT, and adopt proactive measures to minimize resorption risks and optimize treatment outcomes.

## Supplementary Information

Additional file 1.

## Data Availability

The datasets used and/or analysed during the current study are available from the corresponding author on reasonable request.

## Abbreviations

OIRR: Orthodontically induced root resorption
EARR: External apical root resorption
RFT: Root-filled teeth
VPT: Vital pulp teeth
CBCT: Cone-beam computed tomography
3D: Three-dimensional
ROI: Region of interest
dOIRR: Difference in OIRR
M-CSF: Macrophage colony stimulating factor
RANKL: Receptor activator of nuclear factor kappa B ligand
EDTA: Ethylenediaminetetraacetic acid
