# Supplementary material for: Orthodontically induced root resorption in endodontically treated and vital teeth: a cone beam computer tomographic study
Source: Prog Orthod. 2025 Feb 27;26:8. doi: 10.1186/s40510-025-00553-7 (PMC11865387; doi:10.1186/s40510-025-00553-7)
Supplement: Supplementary file 1 — Additional file 1. [file 40510_2025_553_MOESM1_ESM.docx]

**Supplement figure 1.** A heat map of OIRR and root apex movement distance during the orthodontic treatment of each patient included in the research. Vertical axis, the root sample from 1 to 69 clustering according to tooth type; horizontal axis, the studying group (RFT group and VPT group); Gradient solid circle from red to blue, relative value of OIRR; red solid circle, relative value of OIRR from 0 to 3; blue solid circle, relative value of OIRR from -1 to 0; smallest circle, root apex movement distance from 0 mm to 2 mm; middle size circle, root apex movement distance from 2 mm to 4 mm; biggest circle, root apex movement distance from 4 mm to 6 mm.

**Supplement figure 2.** Orthodontic treatment with tooth extraction are associated with longer root moving distances. A, the RFT group; B, the VPT group.
